# Supplementary material for: Magnonic spontaneous oscillation induced by parametric pumping
Source: Nat Commun. 2026 Jul 7;17:5918. doi: 10.1038/s41467-026-71916-9 (PMC13338273; doi:10.1038/s41467-026-71916-9)
Supplement: Supplementary file 1 — Supplementary Information [file 41467_2026_71916_MOESM1_ESM.pdf]

Supplementary Information for

Magnonic spontaneous oscillation induced by parametric pumping

Yi Li, Carissa Kiehl, Jinho Lim, Cliff Abbott, Pratap Kumar Pal, Alex J. Szymczak, Juliang Li, Ralu Divan, Clarence L, Chang, Charudatta Phatak, Dmytro Bozhko, Axel Hoffmann, and Valentine Novosad

Outline

1. **Power dependence of magnon transmission spectrum measured by VNA**
2. **Magnon spontaneous mode at $\mu_0 H_B = 0.21$ T**
3. **Calculation of spontaneous mode output power**
4. **Theoretical calculation of 4-wave mixing conditions**
5. **Identification of probe signal in magnon parametric amplification**

1. Power dependence of magnon transmission spectrum measured by VNA

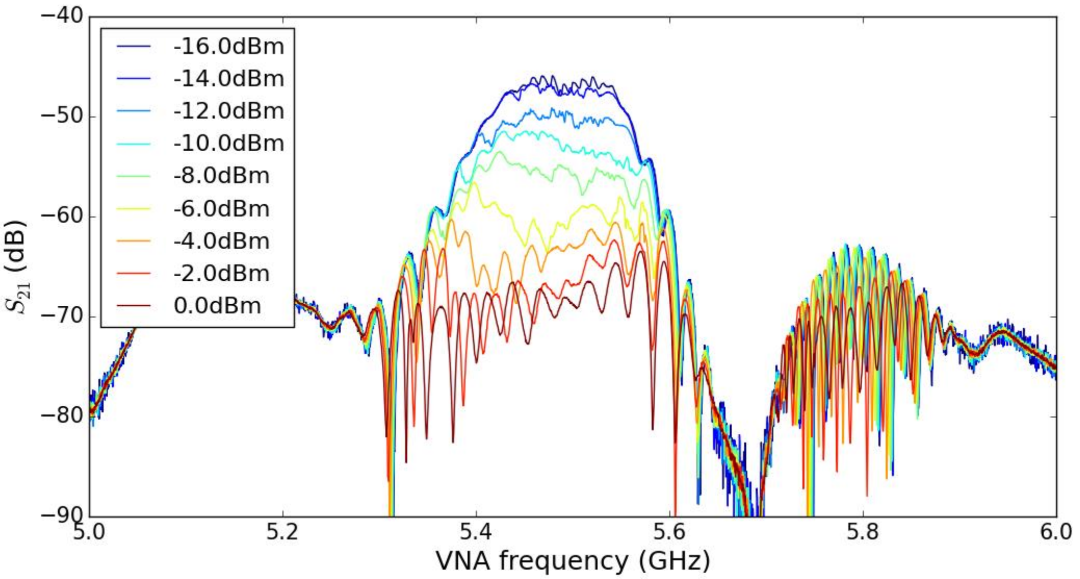

Figure S1 VNA-measured transmission spectra measured at $\boldsymbol{\mu_0 H_B = 0.11}$ **T**, with input power varying from -16 dBm to 0 dBm.

Figure S1 shows the power dependence of magnon transmission spectrum ($\boldsymbol{S_{21}}$) at $\mu_0 H_B = 0.11$ T, corresponding to the experimental data in Figures 1 & 2 of the main text. It can be shown that the magnon transmission band starts to be distorted around $P_{pump} = -14 \sim -12$ dBm, which is where the spontaneous mode starts to show up in Fig. 1(g) of the main text ($P_{pump} = -12.5$ dBm).

Note that the transmission band lineshape is slightly different from the measurement in Fig. 1(c) of the main text. This is because the two experiments were conducted after the sample was unmounted and remounted, causing a different microwave transmission background. Throughout the paper, small peak or band shift may happen owing to slightly different measurement condition, but this does not undermine the robustness of the observed effects.

2. Magnon spontaneous mode at $\boldsymbol{\mu_0 H_B = 0.21}$ T

Here we show the magnon spontaneous mode generated by 4WM at $\mu_0 H_B = 0.21$ T, including the spontaneous mode at $f_{pump} = 8.51$ GHz and $P_{pump} = -10.7$ dBm, which are the main condition for phase locking measurements in Fig. 3 and magnon parametric amplification in Fig. 4 of the main text. Shown in Fig. S1, similar vector network analyzer (VNA) and spectral analyzer (SA) measurements are plotted as in Fig. 1 & 2 of the main text for $\mu_0 H_B = 0.11$ T. This confirms the robustness of the spontaneous mode excitation in a broad frequency band which is tunable by changing either the magnetic field (H_B) or the pump frequency (f_{pump}).

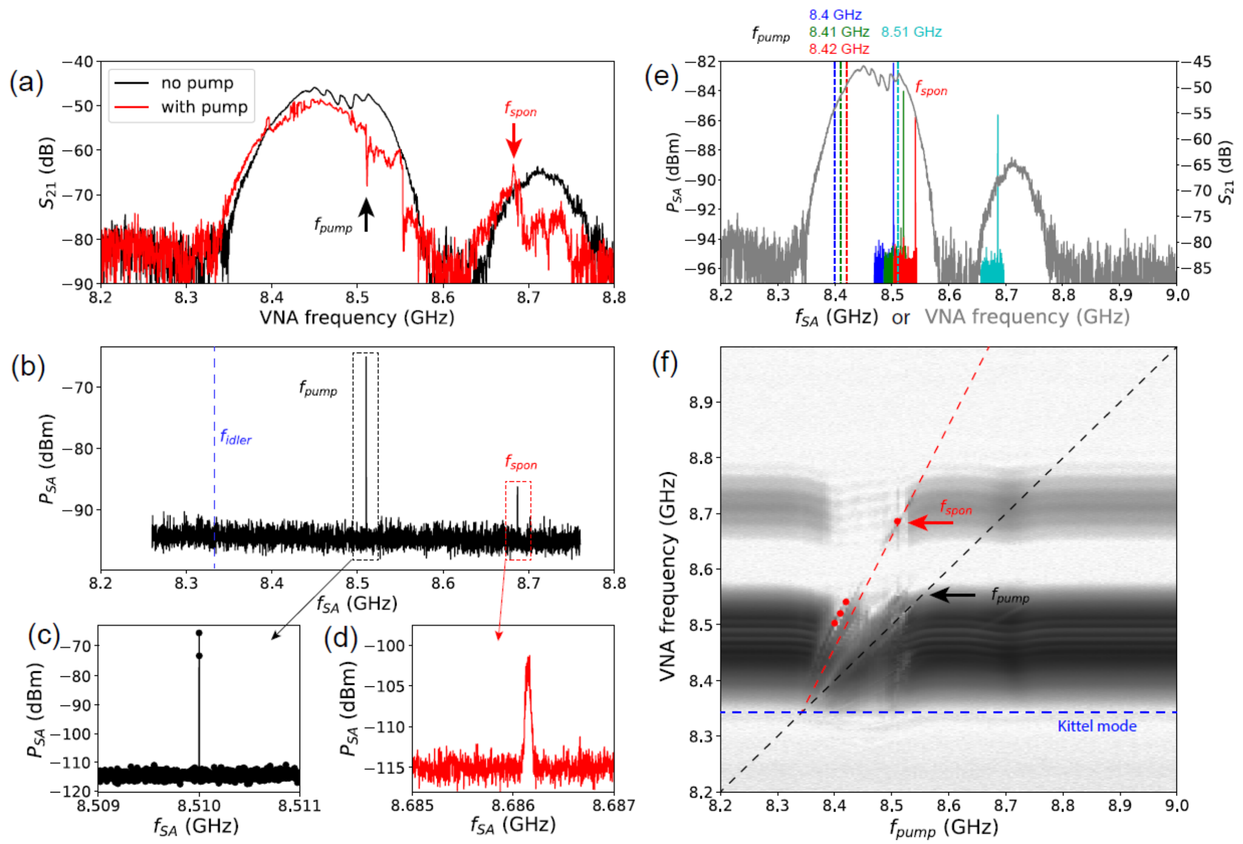

Figure S2 Magnon spontaneous mode measurements at $\mu_0 H_B = 0.21$ **T.** (a) Comparison of VNA measurements with pump on and off at $f_{pump} = 8.51$ GHz and $P_{pump} = -10.7$ dBm. The red arrow indicates the onset of 4WM and generation of the magnon spontaneous mode. (b) SA measurement of spontaneous mode at $f_{pump} = 8.51$ GHz and $P_{pump} = -10.7$ dBm, with a RBW of 20 kHz. (c-d) Zoom-in measurements of (c) the pump signal f_{pump} and (d) the spontaneous mode signal f_{spon} with a RBW of 200 Hz. (e) Evolution of spontaneous mode peak (solid curves) at different f_{pump} (8.4 GHz, 8.41 GHz, 8.42 GHz and 8.51 GHz, shown in dashed vertical lines), with the VNA-measured magnon transmission band (gray curve) plotted for comparison. (f) VNA pump-probe measurements as a function of f_{pump} at $P_{pump} = -10.7$ dBm. Red circles labels SA-measured f_{spon} as a function of f_{pump}.

Note that there is a small frequency drift of the spontaneous mode in Fig. S1(d) (8.6861 GHz) as compared with Fig. 3 (8.698 GHz) and Fig. 4 (8.6858 GHz). The difference between Fig. S1(d) and Fig. 3 is mainly due to the magnetic field hysteresis of the electromagnet. Comparing Fig. S1(d) and Fig. 4, both data were taken under the same magnetic field condition without modifying the field. The small drift (0.3 MHz in this case) is commonly observed throughout the magnon spontaneous mode measurements, and is likely due to the environmental temperature. If we attribute the drift to the magnetic field, then a drift of 0.3 MHz will correspond to a field drift of 0.3 MHz/(2.8 MHz/Oe) = 0.1 Oe taking a gyromagnetic ration of 2.8 MHz/Oe, which may also happen in our experimental environment.

3. Calculation of spontaneous mode output power

The output power of the magnon spontaneous mode can be calculated by integrating the power density throughout the bandwidth, plus considering the resolution bandwidth (RBW) of the

spectral analyzer (SA) measurements. The total power output of SA measurement is calculated by the following equation:

$$P_{out} = P_{SA} \times \Delta f/\mathrm{RBW}\ (\Delta f \gg \mathrm{RBW}) \qquad \text{(S1)}$$

$$P_{out} = P_{SA}\ (\Delta f \ll \mathrm{RBW}) \qquad \text{(S2)}$$

where P_{SA} is the power output for each frequency that is measured within the RBW, Δf is the full-width-half-maximum linewidth of the output signal. For the magnon spontaneous mode, since $\Delta f \gg$ RBW, we will use Eq. (S1) to calculate P_{out}. On the other hand, for the transmitted power from the CW microwave excitation at f_{pump}, the signal linewidth is less than 1 Hz so $\Delta f \ll$ RBW and Eq. (S2) will be used.

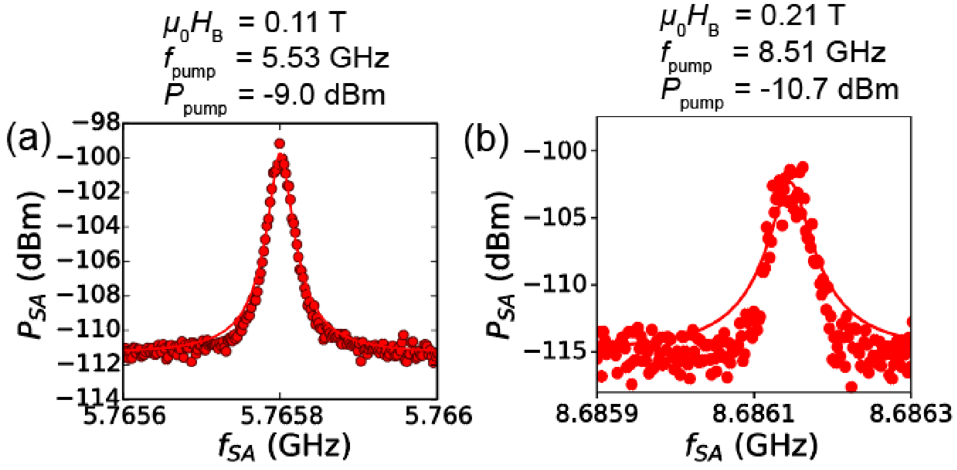

Figure S3 Fits for magnon spontaneous mode measured at two different conditions. (a) is the same as Fig. 1(f). (b) is the same as Fig. S1(d). Curves are fits to a Lorentzian function to the absolute power ($10^{P_{SA}/10}$ mW).

The above calculation can be understood from the SA data in Fig. 1(d), (e) and (f) of the main text at $\mu_0 H_B = 0.11$ T, where Fig. 1(d) is measured at RBW = 20 kHz and Fig. 1(e) and (f) are measured at RBW = 200 Hz. For the main excitation at $f_{pump} = 5.53$ GHz and $P_{pump} = -9.0$ dBm, the peak amplitude remains at $P_{out}^{pump} = -66.2$ dBm for different RBW and thus reflects the true output power. However, both the noise background and the spontaneous mode amplitude are decreased by ~20 dB from Fig. 1(d) to Fig. 1(f), owing to the change of RBW. Note that the spontaneous mode amplitude change is 15 dB, mainly because the mode linewidth $\Delta f = 23.5$ kHz is close to RBW in Fig. 1(d) and the peak amplitude starts to saturate. From Fig. 1(f), with the peak amplitude as $P_{SA} = -100$ dBm, we can calculate the output power of the spontaneous mode from Eq. (S1) as $P_{out}^{spon} = -100\ \mathrm{dBm} + 10\log\left(\frac{\Delta f}{\mathrm{RBW}}\right) = -79.3$ dBm, which is 13.1 dB weaker (5%) than P_{out}^{pump}. This is a very high energy conversion efficiency for magnon spontaneous mode excitation.

For Fig. S1(b-d) measured at $\mu_0 H_B = 0.21$ T, we can also calculate the power conversion efficiency. The main excitation at $f_{pump} = 8.51$ GHz and $P_{pump} = -10.7$ dBm shows a peak amplitude of $P_{out}^{pump} = -65.1$ dBm, close to the power at the condition above. From Fig. S1(d), we extract $P_{SA} = -102.6$ dBm and $\Delta f = 38.4$ kHz, yielding $P_{out}^{spon} = -102.6\ \mathrm{dBm} + 10\log\left(\frac{\Delta f}{\mathrm{RBW}}\right) = -79.7$ dBm, which is 14.7 dB weaker (3.4%) than P_{out}^{pump}. Thus we obtain a quite

consistent spontaneous mode output power and energy conversion efficiency for the magnon four-wave mixing process.

4. Theoretical calculation of 4-wave mixing (4WM) conditions

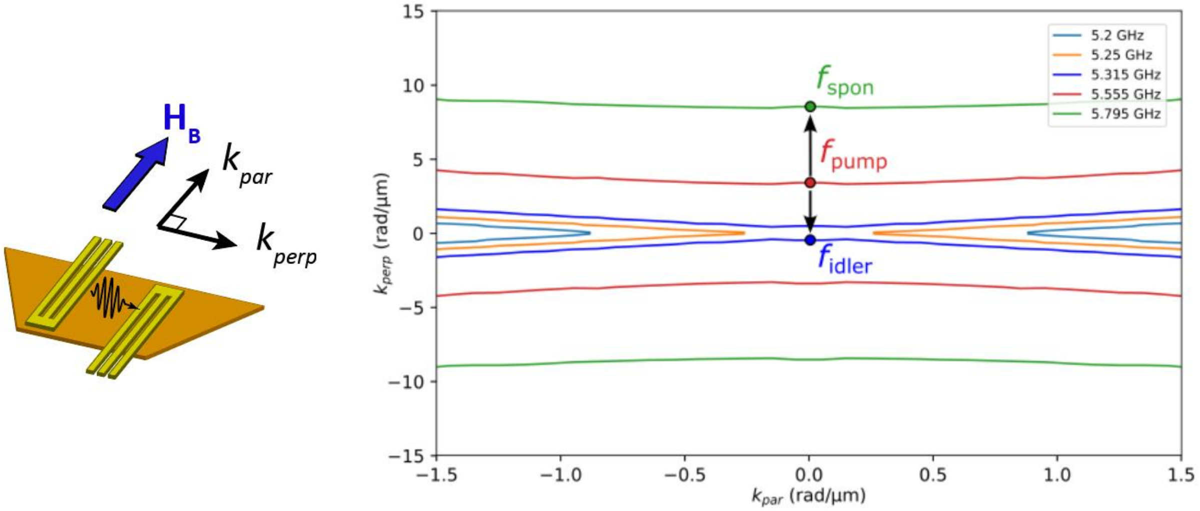

Figure S4 Left: orientation definition of k_{perp} and k_{par} for isofrequency calculation. Right: Theoretical calculation of spin wave isofrequency dispersion curves at $\boldsymbol{\mu_0 H_B = 0.12}$ **T**. showing the condition for 4WM. Here we take pump as $f_{pump} = 5.555$ GHz (orange), spontaneous mode as $f_{spon} = 5.795$ GHz (green), and idler mode $f_{idler} = 5.315$ GHz (blue). The blue and green points mark the condition with wavenumber conservation for k_{perp}, with $k_{par} = 0$.

To evaluate the wavenumber conservation condition in 4WM, we calculate the magnon frequency distribution with isofrequency curves at $\mu_0 H_B = 0.12$ T. In Figure S4, we choose pump mode as $f_{pump} = 5.555$ GHz (orange), spontaneous mode as $f_{spon} = 5.795$ GHz (orange), and idler mode $f_{idler} = 5.315$ GHz (blue), satisfying $2f_{pump} = f_{spon} + f_{idler}$. The frequency choice is made such that the wavenumbers of the Damon-Eshbach mode ($k_{par} = 0$) satisfies $2k_{pump} = k_{spon} + k_{idler}$. In addition, k_{spon} is about twice of k_{pump}, and k_{idler} is nearly zero, agreeing with the the diagram in Fig. 1(b) in the main text. This condition can be found in a broad range of f_{pump}, and in most cases, k_{idler} is negative. This also explains why the k_{idler} mode cannot be measured by the spectrum analyzer, i.e. it propagates away from the detecting antenna.

We also point out that the idler mode can be sometimes measured by the second antenna, as shown in Fig. 4 of the main text. One possibility is that in the 4WM process there could be magnon modes with a finite k_{par} which satisfy both the energy (f) and momentum (k) conservation laws. This may sometimes lead to idler modes with a positive k_{perp} components which can then propagate to the second antenna. Also a finite k_{par} may lead to f_{idler} below the Kittel mode, which is observed in Figure 2b of the main text.

5. Identification of probe signal in magnon parametric amplification

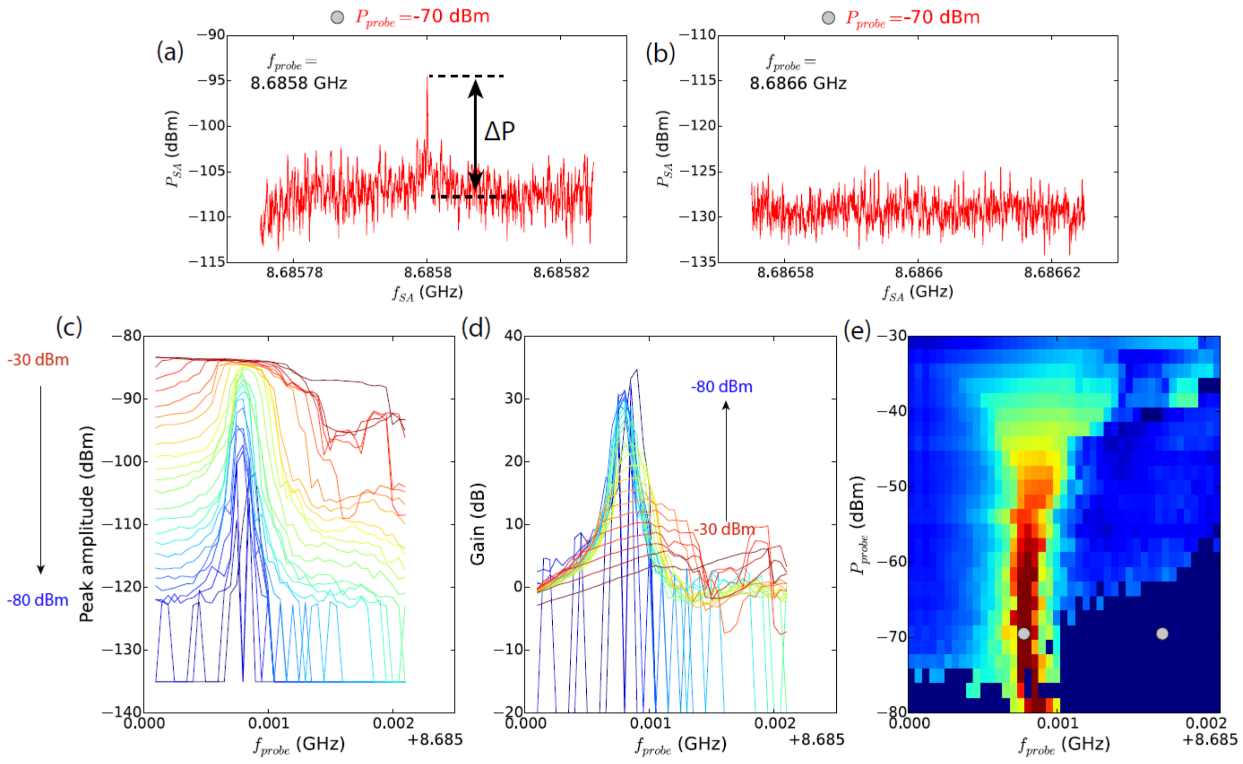

Figure S5 Gain calculation for magnon parametric amplification at $\boldsymbol{\mu_0 H_B = 0.21}$ **T**, $f_{pump} = 8.51$ GHz and $P_{pump} = -10.7$ dBm. (a) Probe tone signal measured at $f_{probe} = 8.6858$ GHz and $P_{probe} = -70$ dBm, showing a sharp peak at f_{probe} above the background. (b) Probe tone signal measured at $f_{probe} = 8.6866$ GHz and $P_{probe} = -70$ dBm, showing no signal at f_{probe}. (c) Extracted peak amplitude of the probe tone as a function of f_{probe} and P_{probe}. If no signal is measured at f_{probe}, the peak amplitude is then set as -135 dBm for eye guidance. (d) Extracted gain from (c). (e) Color plot corresponding to (d), same as Fig. 4(a) of the main text.

Here we explain how probe signals are identified in the magnon parametric amplification experiments. We apply a weak probe microwave signal at f_{probe}, and measure the output spectrum using the spectral analyzer centered at f_{probe}, with a small window of 50 kHz. The RBW is set to 200 Hz. A standard measurement is shown in Figure S5(a), where a sharp peak is measured at $f_{probe} = 8.6858$ GHz. The linewidth is limited by the RBW as 200 Hz, which qualifies the P_{SA} readout as the true output power of the probe signal after amplification.

When the probe power (P_{probe}) is weak, the probe signal may be lost due to the thermal noise. One example is shown in Fig. S5(b), with $f_{probe} = 8.6866$ GHz and $P_{probe} = -70$ dBm, where no clean probe peak is visually observed. In order to identify the amplified probe signal, we set a criteria for the signal:

- *If the peak amplitude at f_{probe} is 5 dB higher than the averaged background [ΔP in Fig. S5(a)], then we take the peak as the probe signal.*

For $\Delta P < 5$ dB, we treat the measurement as "loss of probe signal". In the peak amplitude in Fig. S5(c) we set the amplitude as -135 dBm. In the gain in Fig. S5(d) and (e), we set the gain as -20 dB. This helps to visually show the valid gain measurements in the weak P_{probe} limit.
