# Supplementary material for: Magnonic spontaneous oscillation induced by parametric pumping
Source: Nat Commun. 2026 Jul 7;17:5918. doi: 10.1038/s41467-026-71916-9 (PMC13338273; doi:10.1038/s41467-026-71916-9)
Supplement: Supplementary file 2 — Transparent Peer Review file [file 41467_2026_71916_MOESM2_ESM.pdf]

Peer Review File

Magnonic spontaneous oscillation induced by parametric pumping

Corresponding Author: Dr Yi Li

This file contains all reviewer reports in order by version, followed by all author rebuttals in order by version.

Version 0:

Reviewer comments:

Reviewer #1

(Remarks to the Author)
The manuscript is devoted to experimental study of Suhl instabilities in YIG delay line, as well as phase-locking and parametric amplification operational regimes. The nonlinear nature of the “idler” and “auto-oscillating” modes emergence is clearly demonstrated. A new mechanism of generating magnetic auto-oscillation with broad frequency tunability via parametric pumping is of great scientific and practice interest. The work “Magnonic spontaneous oscillation induced by parametric pumping” looks solid and is well written, and may be accepted for publication after the revision.
Below there are my comments.
1. Equations (1) and (2) can be found in [9] and originally deduced for STNO with internal damping compensation by spin-polarized current for the lowest mode with k = 0. However, the work examines “idler” and “auto-oscillating” modes arising due parametric instability with k ≠ 0. So, the validity of the use of (1) and (2) should be explicitly stated in the text.
2. I agree with authors that developed “concept is important for novel spin wave logic functionalities based on synchronization physics, and can benefit from the intrinsic spin wave nonreciprocity”. However, the role of surface spin wave nonreciprocity (NR) does not well discussed. In the measurement section, it is indicated that the external magnetic field direction for which the maximum magnon transmission band is observed has been selected. For clarity, it will be better to indicate mutual orientation of the wave vector and the magnetic field for this situation. Consider adding an example of measured spectra for different magnetic field directions at least at supplementary. This will enhance the practical significance of the work. What types of NR were found (amplitude or frequency)? Can amplitude NR lead to frequency NR due to nonisochronicity, which can manifest itself as a result of a nonlinear oscillation regime?
3. Existence of lower pumping power limit for “auto-oscillating” peak observance is explained in the text. However, according to the presented measurements, there is also an upper limit. What is its physical reason?
4. The text lacks the references to classical works on magnetization parametric dynamics. Consider citing original works by for example Suhl.
Hereafter still a few comments
• If possible, indicate the direction of the external magnetic field in Fig. 1a and Fig. 3a because it will benefit the clarity of the work.
• There is no refence in the text to Fig.1(d). When discussing phase locking bandwidth dependency on injected microwave power and oscillation power after equation (2) there is no reference to a Fig.3(f) and 3(h). There should be at least one reference in the text for each figure. What is hprobe and Posc?
• Some abbreviations are defined more than once (e.g., VNA, SA).
• It is better to reformulate the following sentence to avoid the use of repeated commas. «The 2k magnon mode shows strong amplitude up to 5% of the pump power and narrow linewidth down to 23.5 kHz, with a quality factor of 245,000, and …».
• There are no definitions of the quantities k_par and k_per in Figure S4 and the text to it. How do they relate to the quantity k in Fig. 1(b)?
In the caption to Figure S4 and in the text to it, “auto-oscillation mode as f_auto =5.795 GHz (orange)” orange should be replaced with (green).
Is it possible to add 10^3 to the axis labels in Figure S4 so as not to depict so many zeros?
• Perhaps it is not worth introducing the circular frequencies ω at all, which are found only in one place in the text (on page 3). Accordingly, it makes sense to designate the frequencies along the horizontal axes of Figures 5(a) and 5(i) in the same way: f_probe.

Reviewer #2

(Remarks to the Author)
The manuscript by Yi Li et al. presents an experimental study on four spinwave mixing in a thin-film spinwave guide with two microwave antennas. Upon directly driving a spinwave mode and exploiting a four spinwave mixing process, nonlinear generation / amplification / control of another spinwave mode is achieved.

The general idea is not particularly novel, and the authors could/should articulate the novelty aspect of their work better. However, the manuscript presents several results that are very interesting experimental findings and will likely be of importance for the developing field of magnonics.

While the quality of experimental results is mostly very good, their interpretation could benefit from more rigor and depth:

Page 1, right column, top paragraph: The discussion of the "well-defined selecting rules" implies that the manuscript solved the problem and imposed such rules making the 4WM beneficial for applications. It is not quite clear what rules (or perhaps rather constraints?) are implied.
2, left, top: "strictly following the theoretical prediction". Such prediction has not been properly elaborated in the manuscript.
2, left, bottom: The ceiling for P_pump should be briefly explained here.
2, top, right: "Here f_idler is not measured.." but isn't it measured later in text?
2, right, paragraph 2: Is \Delta f_auto the tunability window? Why is the tunability ratio important? How is it increasing at lower f_pump?? And how is it related to auto-oscillations disappearing below the 5.39GHz pump?
2, right, par 2 and Fig.2: How is it possible that the idler mode's frequency falls below the Kittel mode?
2, right, par 2: "We highlight the robustness of this 4WM…". This sentence needs to be better elaborated. f_idler happens to be near k=0 and cannot go lower (in contrast to the data in Fig.2) – how does it make the 4WM process robust? Is it implied that a f-k minimum at k=0 increases the number of possible scattering states within the linewidth?
2, right, par 2: "The narrow linewidth shows that … the selection rule is confined in the weak nonlinearity regime". This sentence needs to be elaborated.
2, right, under "Phase Locking": It's probably not the "second" probe signal, but simply a probe signal (that is in addition to the pump signal).
2, right, bottom and Fig.3: Does the observed mode attraction rigorously prove phase-locking? Please explain and provide references ([9]?). Also the harmonic should be better explained.
Eq.1 is "nice" but it does not fit the entire scope of the data.
Fig.1b: Has the f-k been measured and calculated (Supplemental Section 4 does not seem sufficient / clearly representative)? Do both energy conservation and k conservation of the 4WM process check out?
Fig.3 (and others): Is the phase-locked oscillation within the locking band actually visible in the SA measurements? Please explain.
3, left, mid: "idler mode is not observed most likely due to …" here and later in the Supplemental Section 4. The explanation for the irregular appearance of the idler mode does not seem very solid. In particular (re Supplemental Section 4), would the process not be excited symmetrically for positive and negative k? With the film thickness of only 100nm, this should not be a problem for detecting even in the Damon Eshbach geometry?
3, right, top: "In contrast, our magnon parametric amplification requires only pump…". Please address the presence of thermal magnons.
4, left, mid: "... avoids Joule heating" or reduces it (consider antenna)?
3, left, bottom: "Consequently, the amplified magnon signal does not preserve…". This sentence is in (apparent?) contradiction with the preceding one.
4, left, bottom: If "identification of a robust 4WM condition …" is in fact the main novelty of the manuscript, it needs to be expanded and better discussed. See previous comments.

Besides these results related comments, the manuscript could further be improved by addressing the following aspects:

Page 1, right column, top: "reproductivity" or reproducibility?
1, right, bottom: ".. and can be robustly measured (perhaps 'generated' here?) in a broad frequency or (perhaps 'and' here?) external magnetic field…"
1, left, top: "to a second probe signal". Isn't it a second signal, but actually the first probe signal? Same for the abstract.
1, left, top: the concept of the idler mode should be explained or redescribed, instead of simply being dropped in the intro section. The entire sentence is also incomprehensible without having read the rest of the manuscript.
2, left, bottom: "Its existence can be… in the presence of an additional pump signal". The logic of this sentence is unclear.
2, left, bottom: "another idler mode". Perhaps "another mode – i.e. idler mode – …"?
3, left, bottom: explain "clearly compensated frequency shifts"
3, left bottom: "such difference" - unclear choice of the wording.
It would be great to see in all plots additional horizontal or vertical lines that represent the pertinent complementary frequency of the 4WM process (pump or probe or idler etc).
3, right, mid: "constant increase in output power". 'Constant' may not be the best choice of wording here.
The term "phase autonomous" is precise but should be introduced early in the paper for the general reader.
3, right, bottom: Sentence "A maximum gain of 40 dB …" would benefit from rewriting.
4, right, top: "magnon band structure". Perhaps "magnon transmission bands"?
4, left, bottom: "reports of spin-torque amplification.." – are these refs truly spin-torque or effectively a thermal torque? E.g.

[Nat. Commun. 8, 117 (2017)] is strictly speaking also a 4WM. There have also been reports of non-degenerate 3WM with amplification, as in [Phys. Rev. Appl. 11, 034046 (2019)].
4, right, bottom: “is set that a higher”, perhaps “such that”? Also what is sub-microwave CPW?
The entire manuscript could benefit from minor adjustments making it better suited for general readership. There are also a few instances of incorrect grammar, choice of preposition, missing words, etc.
Fig.1 caption: instead of “photon”, “magnon”?

Version 1:

Reviewer comments:

Reviewer #1

(Remarks to the Author)
Review to the updated manuscript
“Magnonic spontaneous oscillation induced by parametric pumping”

The authors concerned most of my comments and submitted a revised version of the manuscript. Generally, I am satisfied with answers. However, there are a number of issues listed below:
1. The explanation provided by authors at the end of «Phase Locking» in new version of manuscript seems a bit confusing. Authors says «If the pump spin wave is filtered, the 4WM-generated mode will be a normal spin wave and lose the phase adaptibility». If the pumping is filtered, i.e., eliminated, then how can nonlinear spin waves occur in the sample at all?
2. In my review, I explicitly asked for an explanation of what hprobe and Posc are. However, hprobe is still not explained in the text and is present in the caption to Fig.3.
3. Figure S4 still lacks clarity. The authors did not replace fauto with fspon. If the isofrequency curves for fpump are shown in orange, as follows from the caption, then why is the fpump icon yellow and lies on the red curve?
4. There is still no reference to Fig.2 (a).
I would like to recommend the paper "Magnonic Spontaneous Oscillations Induced by Parametric Pumping" by Yi Li et al. for publication after minor revisions and corrections.

Reviewer #2

(Remarks to the Author)

Version 2:

Reviewer comments:

Reviewer #1

(Remarks to the Author)
The authors took into account my comments and made appropriate changes to the text. I believe that the work can be recommended for publication.

Open Access This Peer Review File is licensed under a Creative Commons Attribution 4.0 International License, which permits use, sharing, adaptation, distribution and reproduction in any medium or format, as long as you give appropriate credit to the original author(s) and the source, provide a link to the Creative Commons license, and indicate if changes were made.
In cases where reviewers are anonymous, credit should be given to 'Anonymous Referee' and the source.
The images or other third party material in this Peer Review File are included in the article's Creative Commons license, unless indicated otherwise in a credit line to the material. If material is not included in the article's Creative Commons license and your intended use is not permitted by statutory regulation or exceeds the permitted use, you will need to obtain permission directly from the copyright holder.
To view a copy of this license, visit https://creativecommons.org/licenses/by/4.0/

Author response: manuscript NCOMMS-24-62165-T
Magnonic spontaneous oscillation induced by parametric pumping

Yi Li, Carissa Kiehl, Jinho Lim, Cliff Abbott, Pratap K. Pal, Alex J. Szymczak, Juliang Li, Ralu Divan, Clarence L. Chang, Charudatta Phatak, Dmytro Bozhko, Axel Hoffmann, and Valentine Novosad

January 15, 2026

Novelty of our work:

Besides the positive comments from both reviewers, we come aware that both reviewers have questions about the novelty of the work and the clarification of the observed "auto-oscillation" phenomena. We now have a new understanding of the physics behind. The term "auto-oscillation" may be inaccurate. It is a spontaneous spin wave excitation generated by 4-wave mixing, but in an environment of a strong spin wave generated by the pump signal. The nonlinear interaction between these two spin waves, or the parametric instability as suggested by the reviewer, induces an interaction which resembles an auto-oscillator.

Based on the above understanding, we emphasize the main novelty of our work, as:

- 1. We identify a robust 4-wave mixing condition, i.e. two $1k$ magnons are scattered to one quasi-$2k$ magnon and one quasi-Kittel magnon mode, which can generate spontaneous spin wave modes in a broad frequency range and with continuous frequency tunability.
- 2. We show that the spontaneous magnon mode can perform phase-locking and parametric amplifications, which opens a new avenue for studying nonlinear magnonics and synchronization physics in propagating magnon geometry.

Thus, the nature of the magnon spontaneous mode serve as the main novelty of our work. To emphasize the novelty, we have extensively revised the manuscript. We have changed all the terms "magnon auto-oscillation mode" to "magnon spontaneous mode", including the figures and the Supplemental Materials. We have changed all f_{auto} to f_{spon}. We have also changed many languages, which are noted following the reviewers' reports below.

Report of Reviewer 1:

⌈ The manuscript is devoted to experimental study of Suhl instabilities in YIG delay line, as well as phase-locking and parametric amplification operational regimes. The nonlinear nature of the idler and auto-oscillating modes emergence is clearly demonstrated. A new mechanism of generating magnetic auto-oscillation with broad frequency tunability via parametric pumping is of great scientific and practice interest. The work Magnonic spontaneous oscillation induced by parametric pumping looks solid and is well written, and may be accepted for publication after the revision.⌋

We sincerely thank the reviewer for acknowledging the scientific innovation and practical interest of our work.

⌈ 1. Equations (1) and (2) can be found in [9] and originally deduced for STNO with internal damping compensation by spin-polarized current for the lowest mode with $k = 0$. However, the work examines idler

and auto-oscillating modes arising due parametric instability with $k! = 0$. So, the validity of the use of (1) and (2) should be explicitly stated in the text.⌋

We thank the reviewer for the question. To answer the question from the reviewer, the natures of the STNO dynamics and the 4WM magnon spontaneous mode are different, but the resulting dynamic interactions with external probe microwave are the same. Thus, we think the use of the mathematic equations in (1) and (2) is appropriate, but the interpretation needs to be revised to avoid confusion.

In our revised manuscript, we have changed the text at the end of the section "Phase lock" to below:

"The results show that the 4WM-generated magnon mode behaves as an auto-oscillator and can adapt its phase to an external perturbation. However, we point out that this phase adaptibility comes from the parametric instability created by the pumping spin wave. If the pump spin wave is filtered, the 4WM-generated mode will be a normal spin wave and lose the phase adaptibility."

We also changed the description right before Eq. (1) to:

"They can be fitted to the model of a phase auto-oscillator:"

⌈ 2. I agree with authors that developed concept is important for novel spin wave logic functionalities based on synchronization physics, and can benefit from the intrinsic spin wave nonreciprocity. However, the role of surface spin wave nonreciprocity (NR) does not well discussed. In the measurement section, it is indicated that the external magnetic field direction for which the maximum magnon transmission band is observed has been selected. For clarity, it will be better to indicate mutual orientation of the wave vector and the magnetic field for this situation. Consider adding an example of measured spectra for different magnetic field directions at least at supplementary. This will enhance the practical significance of the work. What types of NR were found (amplitude or frequency)? Can amplitude NR lead to frequency NR due to nonisochronicity, which can manifest itself as a result of a nonlinear oscillation regime?⌋

We thank the reviewer for the constructive question. The spin wave nonreciprocity has been previous reported in Ref. [48] [Y. Li et al. Appl. Phys. Lett. 123, 022406 (2023)], and this work used the same device. In Ref. [48], we reported a spin wave nonreciprocity up to 30 dB for the Damon-Eshbach configuration, which was used in this work as well. The NR takes place in the amplitude, and there is no frequency NR for the Damon-Eshbach configuration. Experimentally, we tried to measure the 4WM effect by reversing the spin wave injection direction, and we did not measure any signals in the spectrum analyzer besides the main pump tone.

In our revised manuscript, we have labeled the field and wave vector orientations in Figs. 1(a) and 3(a), and added descriptions to explain the nonreciprocal spin wave excitation under the ge-

ometry. In the section "Magnon spontaneous mode" (previously "Magnon auto-oscillation"), we changed the field orientation description to:

"The magnetic field H_B is parallel to the CPWs and perpendicular to the spin wave wave vector k, as labeled in Fig. 1(a), so that the Damon-Eshbach (DE) propagating magnon mode is excited. Note that under this configuration the spin wave excitation is nonreciprocal, and we take the configuration with a high transmission; see Ref. [48] for more details about spin wave nonreciprocity from the same device."

⌈ 3. Existence of lower pumping power limit for auto-oscillating peak observance is explained in the text. However, according to the presented measurements, there is also an upper limit. What is its physical reason?⌋

The upper limit happens when the pump-induced magnon nonlinearity is too large, leading to a large spin wave dispersion curve distortion such that the 4-wave mixing condition is no longer satisfied.

In our revised manuscript, we added the explanation in the section "Magnon spontaneous mode" (previously "Magnon auto-oscillation") below:

"The upper bound happens when the pump signal induces a large distortion of the spin wave dispersion curve in Fig. 1(b) such that the 4-wave mixing condition is no longer satisfied."

⌈ 4. The text lacks the references to classical works on magnetization parametric dynamics. Consider citing original works by for example Suhl.⌋

We thank the reviewer for the suggestion. In our revised manuscript, we have added the reference to Suhl's classical works in page 1, paragraph 2:

"The process is primarily achieved through Suhl instability [30, 31] in terms of..."

[30] P. W. Anderson and H. Suhl, Phys. Rev. 100, 1788 (1955).

[31] H. Suhl, J. Phys. Chem. Solids 1, 209 (1957).

⌈ Hereafter still a few comments.
If possible, indicate the direction of the external magnetic field in Fig. 1a and Fig. 3a because it will benefit the clarity of the work.⌋

We thank the reviewer for the suggestion. In the revised manuscript, we have added the direction label of the external magnetic field in Fig. 1a and 3a.

⌈ There is no refence in the text to Fig.1(d). When discussing phase locking bandwidth dependency on injected microwave power and oscillation power after equation (2) there is no reference to a Fig.3(f) and 3(h). There should be at least one reference in the text for each figure. What is hprobe and Posc?⌋

In page 2, section "Magnon spontaneous mode" (previously "Magnon auto-oscillation"), we have added the reference to Fig. 1(d):

"...two output peaks can be measured using a spectrum analyzer (SA) as shown in Fig. 1(d):..."

In page 3, we have revised the discussion after Eq. (2) and replaced "hprobe" and "Posc" to be consistent:

"The extracted Δ indeed follows the linear power dependence of $\sqrt{P_{probe}}$ and $1/\sqrt{P_{spon}}$ dependence, as shown by the nice match to the fitting curves in Figs. 3(f) and (h), respectively..."

⌈ Some abbreviations are defined more than once (e.g., VNA, SA).⌋

Revised.

⌈ It is better to reformulate the following sentence to avoid the use of repeated commas. The 2k magnon mode shows strong amplitude up to 5% of the pump power and narrow linewidth down to 23.5 kHz, with a quality factor of 245,000, and .⌋

We take the reviewer's suggestion. In the revised manuscript, we have removed the sentence from the introduction section and added the quality factor in the "Magnon spontaneous mode" (previously "Magnon auto-oscillation") section on page 2 to avoid redundancy:

"...The second peak shows a sharp linewidth of $\Delta f = 23.5$ kHz, corresponding to a quality factor of 245,000."

In the same paragraph, we added the power discussion:

..."The integrated power measured by SA is up to 5% of the measured f_{pump} output at $P_{pump} = -9$ dBm, showing a high energy conversion efficiency of the 4WM process; see the Supplemental Materials for calculation [41]."

⌈ There are no definitions of the quantities k_par and k_per in Figure S4 and the text to it. How do they relate to the quantity k in Fig. 1(b)? In the caption to Figure S4 and in the text to it, "auto-oscillation mode as f_auto =5.795 GHz (orange)" orange should be replaced with (green). Is it possible to add 10^3 to

the axis labels in Figure S4 so as not to depict so many zeros?⌋

Figure S4 shows an expanded version of Figure 1(b) in 2-D spin wave profile. Here k_{par} and k_{perp} are the wave vector components parallel and perpendicular to H_B in the Damon-Eshbach spin wave configuration, respectively. In Figure 1(b) k_{par} is set to zero for simplicity.

In the revised manuscript, we have revised Figure S4 accordingly, including color change, unit change to rad/μm to have less zeros, and adding an inset to mark k_{par} and k_{perp} with respect to H_B.

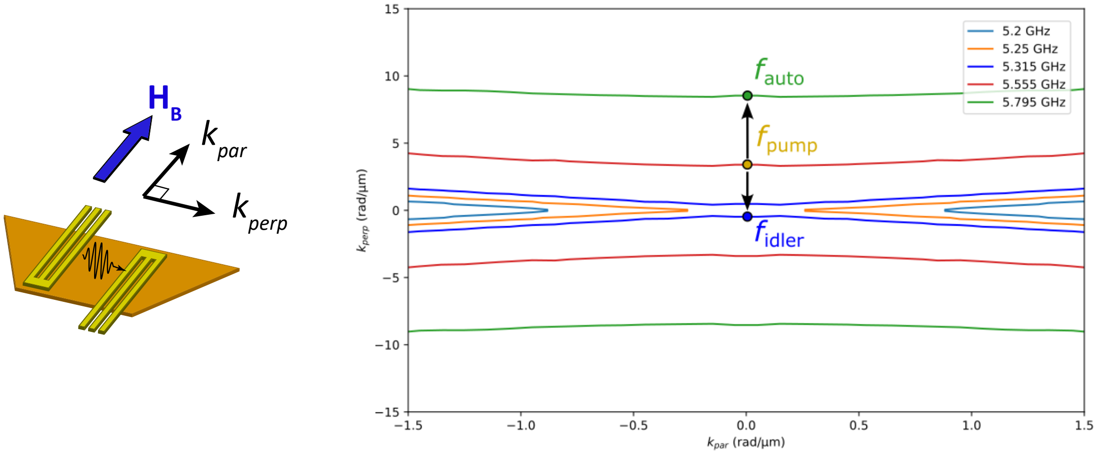

⌈ Perhaps it is not worth introducing the circular frequencies ω at all, which are found only in one place in the text (on page 3). Accordingly, it makes sense to designate the frequencies along the horizontal axes of Figures 5(a) and 5(i) in the same way: f_{probe}.⌋

In the revised manuscript, we adopted the reviewer's suggestion and changed the text in the section "Parametric amplification" as following:

"...both f_{pump} and f_{idler} are needed for amplifying a signal at $2f_{pump} - f_{idler}$."

We also changed the xlabel of Fig. 5(a) from $\omega_{\mathrm{probe}}/2\pi$ to f_{probe}.

Report of Reviewer 2:

⌈ The manuscript by Yi Li et al. presents an experimental study on four spinwave mixing in a thin-film spinwave guide with two microwave antennas. Upon directly driving a spinwave mode and exploiting a

four spinwave mixing process, nonlinear generation / amplification / control of another spinwave mode is achieved.

The general idea is not particularly novel, and the authors could/should articulate the novelty aspect of their work better. However, the manuscript presents several results that are very interesting experimental findings and will likely be of importance for the developing field of magnonics.⌋

We thank the reviewer for the positive comments as well as the question about the novelty of the work. The main novelty of our work are twofold:

- 1. We identify a robust 4-wave mixing condition, i.e. two $1k$ magnons are scattered to one quasi-$2k$ magnon and one quasi-zero-k magnon mode, which can generate spontaneous $2k$ spin wave modes in a broad frequency range and with continuous frequency tunability.
- 2. We show that the spontaneous magnon mode can perform phase-locking and parametric amplifications, which opens a new avenue for studying nonlinear magnonics and synchronization physics in propagating magnon geometry.

Typically magnonic 4-wave mixing are thought to only create chaotic magnon excitations with broad spectral bandwidth or linewidths. Our work show that 4-wave mixing can be utilized in a highly controllable way, along with ultra-narrow spectral linewidth and electrical detection. Furthermore, we show interesting nonlinear magnonic functionality such as phase-locking and parametric amplification, which brings new potential for spin-wave-based microwave information processing.

Below we address the comments about novelty articulation, and many detailed explanation of the phenomena.

⌈ Page 1, right column, top paragraph: The discussion of the "well-defined selecting rules" implies that the manuscript solved the problem and imposed such rules making the 4WM beneficial for applications. It is not quite clear what rules (or perhaps rather constraints?) are implied.⌋

We thank reviewer 2 for the comments. As discussed above, we identify a robust 4-wave mixing condition from $1k$ to quasi-$2k$ and quasi-$0k$. But we agree that this is still not a "well-defined selecting rules" because the quasi-$2k$ and quasi-$0k$ spin wave modes are not quantitatively determined. In the revised manuscript, we have removed the text "well-defined selecting rules",and kept the discussion that the previous 4WM experiments *"lead to lack of controllability and reproducibility for applications."*

⌈ 2, left, top: "strictly following the theoretical prediction". Such prediction has not been properly elaborated in the manuscript.⌋

In the revised manuscript, we have deleted the sentence to avoid redundant discussion. The same point has been discussed in the section "Phase-locking" right after Eq. (2).

⌈ 2, left, bottom: The ceiling for P_{pump} should be briefly explained here.⌋

We thank both reviewers 1,2 for the question. In the revised manuscript, we have added the explanation as discussed in the rebuttal letter to reviewer 1:

The upper bound happens when the pump signal induces a large distortion of the spin wave dispersion curve in Fig. 1(b) such that the 4-wave mixing condition is no longer satisfied.

⌈ 2, top, right: "Here f_{idler} is not measured.." but isnt it measured later in text?⌋

We thank the reviewer for pointing out the issue. In our revised manuscript, we have revised the discussion to:

"We note that f_{idler} does not appear in the SA spectrum in Fig. 1(d). We will show evidence of f_{idler} mode later in the paper."

⌈ 2, right, paragraph 2: Is Δf_{auto} the tunability window? Why is the tunability ratio important? How is it increasing at lower f_{pump}?? And how is it related to auto-oscillations disappearing below the 5.39GHz pump?⌋

We thank the reviewer for the sequential questions. In the revised manuscript, we have changed the way of discussion, focusing now on the 4WM creating a quasi-$2k$ mode and a quasi-Kittel mode. We have removed the discussion of tunability ratio because it is highly sensitive to the DE spin wave dispersion curve and there is no easy analytical solution. Instead, we compare the f_{spon} and f_{idler} modes to the $2k$ mode in red dashed line and the Kittel mode in blue dashed line, as shown in the updated Figure 2. Figure S2 has been updated likewise.

We have changed the discussion to the following:

"In addition, we plot the expected $f_{idler} = 2f_{pump} - f_{spon}$ in blue stars. The location of f_{idler} is close to the Kittel mode (f_K, $k = 0$) as shown the in blue dashed line, and f_{spon} is close to $2f_{pump} - f_K$ as shown in the red dashed line. Note that because the spin wave wavelengths are above 1 μm, the DE mode has a quasi-linear f-k dispersion curve, Thus, $2f_{pump} - f_K$ is a good approximation of the spin wave mode with twice the wavenumber as the f_{pump} mode. The comparisons show that the 4WM process converts two f_{pump} magnons with wavenumber k to one quasi-Kittel mode with nearly zero wavenumber and another spin wave mode with a wavenumber close to $2k$. We highlight the robustness of the 4WM process in a broad frequency range. The upper frequency bound is limited by the CPW antenna bandwidth. The lower frequency bound is cause by the reduced

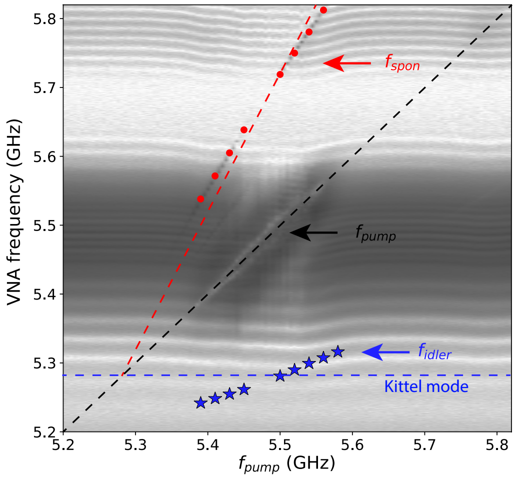

pump efficiency as f_{pump} starts to move out of the antenna transmission band."

⌈ 2, right, par 2 and Fig.2: How is it possible that the idler mode's frequency falls below the Kittel mode?⌋

We thank the reviewer for the question, as we are also a bit surprised about it. One possibility is that the 4WM involves spin wave modes with finite k_{par}, as plotted in Figure S4 of the Supplemental materials. With a wavevector component parallel to the magnetization (i.e. parallel to the antenna electrodes), the spin wave mode will contain a backward-volume component, which will lower the frequency even below the Kittel mode.

In the resubmission, we have included the discussion in the Supplemental Materials with Figure S4. The quantitative discussion of the 4WM condition is beyond the scope of this paper and thus we only provide qualitative discussion to cover the possibilities.

"...Also a finite k_{par} may lead to f_{idler} below the Kittel mode, which is observed in Figure 2b of the main text."

⌈ 2, right, par 2: "We highlight the robustness of this 4WM...". This sentence needs to be better elaborated. f_{idler} happens to be near k=0 and cannot go lower (in contrast to the data in Fig.2) how does it make the 4WM process robust? Is it implied that a f-k minimum at k=0 increases the number of possible scattering states within the linewidth?⌋

As explained in the previous comment, f_{idler} may go below the Kittel mode when a finite k_{par} is excited and a finite backward-volume component is present in the f_{idler} mode. The robustness means that 4WM can be observed in a broad f_{pump} and f_{spon} range, making it a tunable spin wave

with a decent tuning range.

In the revised manuscript, we have changed the sentence to:

"...We highlight that the 4WM-generated f_{spon} mode can be robustly observed in a broad frequency range."

⌈ 2, right, par 2: "The narrow linewidth shows that ... the selection rule is confined in the weak nonlinearity regime". This sentence needs to be elaborated.⌋

The linewidth of the f_{spon} mode is determined by the thermal fluctuation of the YIG delay line device. If there is no thermal fluctuation, there should be only one 4WM condition which exactly satisfies f and k conservations. Due to thermal fluctuation, the 4WM selection rule is expanded to a range of f and k.

In the revised manuscript, we have removed the sentence and modified the related linewidth discussion in the "Discussion" section on page 4 of the manuscript:

"...The linewidth of the f_{spon} mode is determined by the thermal noise of the YIG delay line device, which expands the 4WM selection rule to a finite frequency range."

⌈ 2, right, under "Phase Locking": Its probably not the "second" probe signal, but simply a probe signal (that is in addition to the pump signal).⌋

Yes, as the reviewer suggests, we have changed the text from "...a second probe signal" to *"...a probe signal"*.

⌈ 2, right, bottom and Fig.3: Does the observed mode attraction rigorously prove phase-locking? Please explain and provide references ([9]?). Also the harmonic should be better explained.⌋

Within the phase-locking bandwidth, we observe that the power of the probe tone is increased, which is shown in Fig. 5(a) as a small gain above $P_{\text{probe}} = -40$ dBm. The gain comes from the phase-locking effect, where P_{probe} is strengthened by incorporating the f_{spon} mode. It can be also observed from Fig. 5(a) that the gain is decreased as P_{probe} increases above -40 dBm. This is because the gain is calculated as $(P_{\text{probe}} + P_{\text{spon}})/P_{\text{probe}} = 1 + P_{\text{spon}}/P_{\text{probe}}$. With a constant P_{spon}, increasing P_{probe} will decrease the ratio and thus the gain. The related discussion is provided in the section "Parametric amplification".

The harmonics is a typical behavior in nonlinear microwave system as $f_{\text{harmonic}} = 2f_{\text{spon}} - f_{\text{probe}}$. In the revised manuscript, we have added the following sentence right after Eq. (1):

"The harmonic mode in Figs. 3(c) can be fitted by $f_{harmonic} = 2f_{spon} - f_{probe}$, with the fitting curve also shown in Figs. 3(d)."

⌈ Eq.1 is "nice" but it does not fit the entire scope of the data.⌋

In Fig. 3(d) Eq. (1) fits well to the experimental data in our opinion. If the reviewer is referring to the data in Fig. 3(e), it is usually normal that at large probe power spontaneous dynamics could exhibit chaotic behaviors when f_{probe} is close to f_{spon}, as can be observed in most spin-torque nano-oscillator papers.

⌈ Fig.1b: Has the f-k been measured and calculated (Supplemental Section 4 does not seem sufficient / clearly representative)? Do both energy conservation and k conservation of the 4WM process check out?⌋

We did not calculate f-k dispersion curve here, mainly because the 4WM only happens when the pump magnon mode is excited in the nonlinear regime. Here the f-k dispersion curve will be distorted, as shown in Fig. 2(b) as the VNA transmission band distortion in the vicinity of f_{pump}. Supplemental Section 4 only gives a qualitative example of 4WM condition by ignoring the f-k dispersion curve distortion and taking both energy and momentum conservations.

4WM has to meet both energy and momentum conservation. For example, in Fig. 4(b), (e) and (f), f_{spon} and f_{idler} strictly follows $f_{\text{spon}} + f_{\text{idler}} = 2f_{\text{pump}}$. In Fig. 2(b), the calculation of f_{idler} is based on energy conservation ($f_{\text{idler}} = 2f_{\text{pump}} - f_{\text{spon}}$). We don't know the exact wavenumber of f_{spon} and f_{idler} modes, but their locations are close to $2k$ and Kittel mode ($k = 0$). The analogy helps to understand the momentum conservation in this process.

⌈ Fig.3 (and others): Is the phase-locked oscillation within the locking band actually visible in the SA measurements? Please explain.⌋

Yes the phase-locked oscillation is visible in the SA measurements. One example is in Fig. 5(b-d), where the raw data of SA measurements is shown at three different f_{probe}. Fig. 5(b) shows clearly that only a single peak is present and the magnon spontaneous mode peak, as shown in Fig. 5(e-g), is absorbed into the probe tone.

⌈ 3, left, mid: "idler mode is not observed most likely due to " here and later in the Supplemental Section 4. The explanation for the irregular appearance of the idler mode does not seem very solid. In particular (re Supplemental Section 4), would the process not be excited symmetrically for positive and negative k? With the film thickness of only 100nm, this should not be a problem for detecting even in the Damon Eshbach geometry?⌋

We acknowledge the comment from the reviewer. Indeed the appearance of the idler mode is

irregular. This is mainly due to the limitation in electrical detection. Optical detection such as Brillouin light scattering or MOKE would be a better tool to search for the idler mode. Also due to the complication in nonlinear spin wave band distortion at f_{pump}, it is hard to quantify the 4WM conditions. This is one limitation of our work. But since the main innovation is to reveal such robust 4WM process with narrow linewidths, as well as many interesting physics features such as phase-locking and parametric amplification, we believe our results, even without quantitative analysis of the 4WM condition, should still lead to broad interests in nonlinear magnonics for spin wave information processing.

For the negative k condition, the Damon Eshbach geometry exhibits a strong nonreciprocal spin wave excitation; see our previous work for example (Li, et al. Appl. Phys. Lett. 123, 022406 (2023), Ref. [40]). The spin wave transmission with negative k condition (i.e. reversing the microwave input and output ports or reversing the magnetic field direction) is too weak for observing such effects. If we reverse both the microwave input-output ports and the magnetic field, then we can still observe the same 4WM as well as phase-locking and parametric amplification.

⌈ 3, right, top: "In contrast, our magnon parametric amplification requires only pump". Please address the presence of thermal magnons.⌋

We are not sure how thermal magnons will be involved in the parametric amplification process. The effect of thermal magnons may lead to the linewidth broadening of f_{spon} mode. What is measured by the SA is the power density at each frequency, which is the key for determining the gain of parametric amplification. However, the detailed discussion is beyond the scope of this manuscript as there needs to be a complicated theoretical treatment of such phenomena. We did not find any available physical modeling of such process with the onset of 4WM.

⌈ 4, left, mid: "... avoids Joule heating" or reduces it (consider antenna)?⌋

Changed from "avoids" to "reduces".

⌈ 3, left, bottom: "Consequently, the amplified magnon signal does not preserve". This sentence is in (apparent?) contradiction with the preceding one.⌋

We thank the reviewer for pointing out the confusing discussion. The point we want to express is that, the previous 3WM parametric amplification utilizes the magnon modes at half the pump frequency, which keeps a fixed phase relation with the pump tone. Our work utilizes 4WM process where the f_{spon} mode has no phase correlation to f_{pump}. This means the 4WM parametric amplification process can benefit from the phase-sensitive nonlinear magnon interaction between f_{spon} and the probe tone, whereas for the 3WM parametric amplification the $f_{\mathrm{pump}}/2$ mode cannot have phase-sensitive nonlinear magnon interaction with the probe tone. This is why we can have a large

gain up to 40 dB in the weak probe power limit, where the amplification in the 3WM works may be a result of linear addition of the $f_{\mathrm{pump}}/2$ signal on top of the weak probe tone.

In our revised manuscript, we have changed the text to:

"...In those studies, 3WM generates magnons at half the pump frequency which maintain fixed phase relation with the pump tone. This means the half-frequency magnon mode will not be able to adapt its phase to another external probe tone, and will not lead to parametric amplification with phase-sensitive nonlinear interaction as reported in our work."

⌈ 4, left, bottom: If "identification of a robust 4WM condition " is in fact the main novelty of the manuscript, it needs to be expanded and better discussed. ⌋

Here our main novelty are not only on the robustness of 4WM, but also on the interesting phenomena such as phase-locking and parametric amplification for their potential in studying nonlinear magnonics and developing spin wave information processing. The last paragraph of the "Discussion" section focuses on the comparison of the 4WM process in our work with other reports. The discussion of the second novelty is in the first paragraph of the "Discussion" section.

In the revised manuscript, we have made the following change to the last paragraph to be more explicit about our point:

"The main difference of our work is that we identify a routine for consistent and reliable 4WM with strong power output, narrow linewidth, and broad frequency tunability up to 300 MHz by changing f_{pump} and from 5 to 8 GHz by changing H_B. The tuning range of the magnon spontaneous mode has no theoretical limit and can be extended to any value supported by H_B and the magnon band structure defined by the CPW geometry. This provides a good foundation of bandwidth for future spin wave information processing based on nonlinear magnonics."

⌈ Besides these results related comments, the manuscript could further be improved by addressing the following aspects:
Page 1, right column, top: "reproductivity" or reproducibility? ⌋

Changed.

⌈ 1, right, bottom: ".. and can be robustly measured (perhaps 'generated' here?) in a broad frequency or (perhaps 'and' here?) external magnetic field" ⌋

Changed to "generated" and "and".

⌈ 1, left, top: "to a second probe signal". Isnt it a second signal, but actually the first probe signal? Same for the abstract. ⌋

In the revised manuscript, we have removed all the word "second" when it is referred to the probe signal, as we only use one probe signal in this work.

⌈ 1, left, top: the concept of the idler mode should be explained or redescribed, instead of simply being dropped in the intro section. The entire sentence is also incomprehensible without having read the rest of the manuscript. ⌋
⌈ 2, left, bottom: "Its existence can be in the presence of an additional pump signal". The logic of this sentence is unclear. ⌋
⌈ 2, left, bottom: "another idler mode. Perhaps another mode i.e. idler mode "? ⌋

We take the reviewer's suggestion. In the revised manuscript, we have rewritten the 3rd paragraph for introducing the spontaneous mode and the idler mode in the 4WM process:

In this work, we demonstrate robust excitation of magnon spontaneous oscillations in a yttrium iron garnet (YIG) thin-film delay line. By controlling the pump power to be slightly above the Suhl instability threshold, we trigger a 4WM process which converts the pump spin wave with wavenumber k to a higher-frequency spin wave mode with nearly twice the wavenumber (spontaneous mode) and a lower-frequency spin wave mode with nearly zero wavenumber (idler mode). The spontaneous mode can be electrically detection when it is within the antenna-defined magnon transmission band, and can be generated over a broad frequency range by tuning the pump frequency and the external magnetic field. The idler mode cannot be always measured due to its decoupling with the antenna. We show the existence of the idler mode when the pump frequency is close to the Kittel mode. In addition, we show that the 4WM-generated spontaneous mode can be phase-locked to a probe signal. This proves that the spontaneous mode is phase-autonomous, i.e. it can adapt its phase to external stimulus without being bound to the pump. The idler mode can be phase-locked as well, and a parametric phase-locking of the spontaneous mode is simultaneously observed with the same locking bandwidth, proving their correlation in the 4WM process. Furthermore, we show that at weak probe powers well below the phase-locking regime, the spontaneous mode can act as a magnon parametric amplifier to a weak probe signal with a constant gain that is determined by the amplitude of the oscillator. The results open a new avenue of creating robust and highly controllable nonlinear spin wave dynamics in solid state, and utilizing the nonlinear dynamics in synchronization physics and microwave information processing.

⌈ 3, left, bottom: explain "clearly compensated frequency shifts" ⌋

In the revised manuscript, we have revised the discussion and made it explicit:

"We also observe that within the f_{probe} scanning window, the drifts of f_{spon} and f_{idler} are always compensated such that $f_{autospon} + f_{idler} = 2f_{pump}$ is satisfied."

⌈ 3, left bottom: "such difference" - unclear choice of the wording. ⌋

We notice that the discussion is unclear and redundant. In the revised manuscript, we have removed the part discussing the possibility that *"...the spontaneous mode is phase-locked by the mixing harmonics between the probe and the pump"*.

⌈ It would be great to see in all plots additional horizontal or vertical lines that represent the pertinent complementary frequency of the 4WM process (pump or probe or idler etc). ⌋

In the revised manuscript, we have added a vertical line in Fig. 1(d) to mark f_{idler}, and dashed lines in Fig. 2(b) to compared f_{spon} and f_{idler} with Kittel mode based 4WM conditions.

⌈ 3, right, mid: "constant increase in output power". 'Constant' may not be the best choice of wording here. ⌋

In the revised manuscript, we have changed the wording to:

"...resulting in a linear increment of output power".

⌈ The term "phase autonomous" is precise but should be introduced early in the paper for the general reader. ⌋

In the revised manuscript, we added the definition of "phase autonomous" in the last paragraph of the introduction:

"...In addition, we show that the 4WM-generated spontaneous mode can be phase-locked to a probe signal. This proves that the spontaneous mode is phase-autonomous, i.e. it can adapt its phase to external stimulus without being bound to the pump."

⌈ 3, right, bottom: Sentence "A maximum gain of 40 dB " would benefit from rewriting. ⌋

In the revised manuscript, we have modified the language to *"...A gain up to 40 dB is obtained..."*.

⌈ 4, right, top: "magnon band structure". Perhaps "magnon transmission bands"? ⌋

Changed.

⌈ 4, left, bottom: reports of spin-torque amplification.. are these refs truly spin-torque or effectively a thermal torque? E.g. [Nat. Commun. 8, 117 (2017)] is strictly speaking also a 4WM. There have also been reports of non-degenerate 3WM with amplification, as in [Phys. Rev. Appl. 11, 034046 (2019)]. ⌋

We thank the reviewer for bringing up the questions as well as the two related references. Certainly both spin current injection and thermal spin inject could play a role in dc-current-based spin wave amplification. It is well known that for spin-torque effects such as magnetization switching and auto-oscillation, the thermal effect and spin injection effect cannot be fully disentangled (see examples of antiferrimagnetic switching). The two references are good examples of thermal torque discussion for spin-torque oscillators and spin wave amplifcations.

In our revised manuscript, we have added the reference of Spin caloritronic nano-oscillator [Nat. Commun. 8, 117 (2017)] into the introduction to expand the scope of the auto-oscillator discussion, and the [Phys. Rev. Appl. 11, 034046 (2019)] reference into the discussion of spin-torque amplification of spin wave.

⌈ 4, right, bottom: is set that a higher, perhaps such that? Also what is sub-microwave CPW? ⌋

The first part has been changed to *"...is such that a higher..."*. The second part has been changed to *"...two CPW antennas with sub-micron electrode widths"*

⌈ The entire manuscript could benefit from minor adjustments making it better suited for general readership. There are also a few instances of incorrect grammar, choice of preposition, missing words, etc. ⌋

We thank the reviewer for the suggestion. In the revised manuscript, we have carefully proofread the manuscript and improved the language and grammar.

⌈ Fig.1 caption: instead of photon, magnon? ⌋

Changed from *"photons"* to *"magnons"*.

Author response: manuscript NCOMMS-24-62165-T
Magnonic spontaneous oscillation induced by parametric pumping

Yi Li, Carissa Kiehl, Jinho Lim, Cliff Abbott, Pratap K. Pal, Alex J. Szymczak, Juliang Li, Ralu Divan, Clarence L. Chang, Charudatta Phatak, Dmytro Bozhko, Axel Hoffmann, and Valentine Novosad

February 13, 2026

Report of Reviewer 1:

⌈ 1. The explanation provided by authors at the end of "Phase Locking" in new version of manuscript seems a bit confusing. Authors says "If the pump spin wave is filtered, the 4WM-generated mode will be a normal spin wave and lose the phase adaptibility". If the pumping is filtered, i.e., eliminated, then how can nonlinear spin waves occur in the sample at all? ⌋

We thank the reviewer for pointing out the unclear statement. We agree with the reviewer that the pump spin wave cannot be filtered or eliminated, otherwise nonlinear spin wave interaction will not occur. After examining the context, we have simply removed the sentence to avoid further confusion.

⌈ 2. In my review, I explicitly asked for an explanation of what hprobe and Posc are. However, hprobe is still not explained in the text and is present in the caption to Fig.3. ⌋

We thank the reviewer for pointing out the mistake. In the revised manuscript, we have replaced h_{probe} by $\sqrt{P_{\text{probe}}}$, and h_{probe} no longer shows up in the entire manuscript. To be more specific, Posc is the power of the spontaneous spin wave mode due to 4WM and has been replaced by P_{spon}.

In the revised manuscript, we have added the explicit description of P_{spon} right after Eq. (2):

"..Here P_{probe} is the probe microwave power and P_{spon} is the power of the measured spontaneous spin wave mode."

⌈ 3. Figure S4 still lacks clarity. The authors did not replace fauto with fspon. If the isofrequency curves for fpump are shown in orange, as follows from the caption, then why is the fpump icon yellow and lies on the red curve? ⌋

We thank the reviewer for pointing out the inconsistency. In the revised manuscript, we have correctly replaced fauto with fspon, and changed the color of the isofrequency curves accordingly. The new Figure S4 now looks as below:

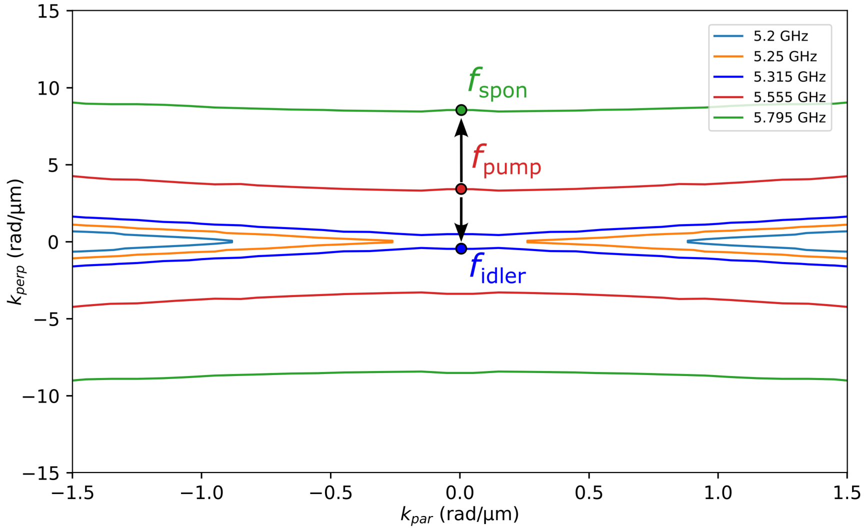

⌈ 4. There is still no reference to Fig.2 (a). ⌋

In our revised manuscript, we have added the reference to Fig. 2(a) as following in the second paragraph of section "Magnon spontaneous mode" on page 2:

"...By changing f_{pump} from 5.50 to 5.58 GHz, the spontaneous mode mode can be detected and f_{spon} changes from 5.7187 to 5.8438 GHz, as shown in 2(a), showing a wide range of frequency tunability."
